# Provider-initiated HIV testing in rural Haiti: low rate of missed opportunities for diagnosis of HIV in a primary care clinic

**DOI:** 10.1186/1742-6405-4-28

**Published:** 2007-11-29

**Authors:** Louise C Ivers, Kenneth A Freedberg, Joia S Mukherjee

**Affiliations:** 1 Department of Medicine, Brigham and Women's Hospital, Harvard Medical School, Boston, USA; 2 Department of Medicine, Massachusetts General Hospital, Harvard Medical School, Boston, USA; 3 Partners In Health, Boston, USA

## Abstract

As HIV treatment is scaled-up in resource-poor settings, the timely identification of persons with HIV infection remains an important challenge. Most people with HIV are unaware of their status, and those who are often present late in the course of their illness. Free-standing voluntary counseling and testing sites often have poor uptake of testing. We aimed to evaluate a 'provider-initiated' HIV testing strategy in a primary care clinic in rural resource-poor Haiti by reviewing the number of visits made to clinic before an HIV test was performed in those who were ultimately found to have HIV infection. In collaboration with the Haitian Ministry of Health, a non-governmental organization (Partners In Health) scaled up HIV care in central Haiti by reinforcing primary care clinics, instituting provider-initiated HIV testing and by providing HIV treatment in the context of primary medical care, free of charge to patients. Among a cohort of people with HIV infection, we assessed retrospectively for delays in or 'missed opportunities' for diagnosis of HIV by the providers in one clinic. Of the first 117 patients diagnosed with HIV in one clinic, 100 (85%) were diagnosed at the first medical encounter. Median delay in diagnosis for the remaining 17 was only 62 days (IQR 19 – 122; range 1 – 272). There was no statistical difference in CD4 cell count between those with and without a delay. 3787 HIV tests were performed in the period reviewed. Provider-initiated testing was associated with high volume uptake of HIV testing and minimal delay between first medical encounter and diagnosis of HIV infection. In scale up of HIV care, provider-initiated HIV testing at primary care clinics can be a successful strategy to identify patients with HIV infection.

## Introduction

Only 5–8% of individuals with Human Immunodeficiency Virus (HIV) infection globally are aware of their diagnosis [1]. In the developing world, early 'in-program' mortality has been prominent in a number of HIV treatment programs, often due to patients presenting late for care with already advanced disease [2]. In the ongoing effort to prevent new HIV infections and to treat those with established infection, emphasis must be placed on developing strategies to effectively identify and engage HIV-infected patients into care, with HIV testing as a critical step.

Much of the HIV testing in the developing world is done through maternity clinics offering antiretroviral drugs for the prevention of maternal to child transmission of HIV or specialty voluntary counseling and testing (VCT) clinics to which people come desiring knowledge of their status. Studies from such settings in South Africa and Côte d'Ivoire have identified factors including 'fear of a positive HIV test', low levels of education and poor housing as associated with low uptake rates [3,4]. US and African studies have identified high rates of "missed opportunities" for making the diagnosis of HIV among primary care clinicians who did not offer HIV tests to their patients even if they had clinical syndromes associated with HIV infection or if they were from a high-risk sociodemographic group [5-10].

In Haiti neither primary health care nor HIV VCT is widely available and the estimated HIV prevalence is estimated between 2.2 and 3.8% [11,12]. In the central plateau department, a 2005 Demographic and Health Survey reported the seroprevalence of HIV in 15–49 year olds as 1.6% [12]. In 2002, when the Global Fund to Fight AIDS, TB and Malaria called for applications, Partners In Health – a non-profit organization affiliated with Harvard Medical School – began a collaborative program with the Haiti Ministry of Health with the goal of improving access to primary care as an avenue to provide HIV prevention, testing and treatment. To achieve this goal, staffing levels were improved for general patient care and non-HIV related essential medicines were provided free of charge to patients seeking care. All health care providers – including doctors, nurses, social workers and community health workers were educated about the signs and symptoms of HIV, the importance of active case finding and contact tracing and the urgency for testing those who appeared ill even if they did not present specifically for HIV testing. To determine whether this approach was effective in identifying patients with HIV in a timely manner, we reviewed the number of clinic visits that a patient had prior to ultimately testing HIV positive in one of our clinics.

## Methods

We performed a retrospective chart review of 117 patients who were ultimately diagnosed with HIV infection attending a public general medical clinic revitalized with monies from the GFATM in Boucan Carré, Haiti, between March 1, 2003 and December 31, 2003. Medical records were reviewed for each HIV-infected patient and the following data were abstracted: age, sex, date of HIV diagnosis, date of first visit to the clinic, number of encounters at the clinic prior to HIV diagnosis, number of days between first visit and diagnosis, and CD4 cell count at time of diagnosis. Any visit to the clinic before the diagnosis of HIV was made (dating back to March 2003) was considered to be a 'missed opportunity' for that patient. Prior to March 2003 the medical clinic was only minimally functional and records were inconsistently kept, HIV testing and treatment and most primary healthcare services were not available (see discussion for further details). 'Delay' was defined as the number of days between the date of the first visit to the clinic and the date that the diagnosis of HIV was ultimately made. HIV testing was performed on plasma specimens using a standardized algorithm of two rapid tests [Determine™ HIV1/HIV2, (Abbott Pharmaceuticals); Capillus™ HIV1/HIV2 (Cambridge Diagnostics Ireland Ltd)] with discordant results settled by Western Blot. Statistical analysis was performed using SAS 9.1™software. We used the Wilcoxon Rank Sum Test to assess differences in CD4 cell count at the time of diagnosis between those with and without a delay in diagnosis, and chi-square analysis for comparison of proportions. This study was approved by Brigham and Women's Hospital Institutional Review Board in USA, and by the Zanmi Lasante Ethics Committee in Haiti.

## Results

During the review period, 3787 HIV tests were performed and 117 patients were newly diagnosed with HIV infection and included in this study (seroprevalence 3.1%). Fifty-five percent of adult visits (N = 6859) resulted in an HIV test being completed. Of 117 patients diagnosed with HIV, data on prior clinical encounters were available for 112. Medical record review suggested that none of the individuals had previously had an HIV test. Seventy percent were female. Median first CD4 cell count was 351 cells/mm^3 ^(Interquartile range 212 – 624; range 49 – 1568 cells/mm^3^). Seven patients (7.0%; 95% Confidence Interval 2.9 – 13.9%) had CD4 cell counts <100 cells/mm^3 ^at diagnosis and twenty-one patients (21%; 95% CI 13.5 – 30.3%) had CD4 cell counts <200 cells/mm^3^at diagnosis. CD4 cell count data were missing for twelve patients.

Ninety-five of the 112 patients (84.8%; 95% CI 76.8 – 90.9%) had their HIV diagnosis made at the time of their first clinic visit. Of the 17 patients not diagnosed on the first visit, 14 (12.5%; 95% CI 7.0 – 20.0%) were tested and diagnosed on the second visit, two (1.8%; 95% CI 0.2 – 6.3%) on the third visit and one (0.9%; 95% CI < 0.1 – 4.9%)) on the fifth visit to the clinic.

Of the 17 who were not diagnosed on the first visit, 14 were female (82%); three were male (18%). The median delay in diagnosis (i.e. the number of days between first clinic visit and HIV diagnosis) was 62 days (IQR 19 – 122; range 1 to 272 days). No patient's diagnosis was delayed as a result of refusing an HIV test. There was no significant difference in median CD4 cell count between those patients who had any delay in diagnosis (447 cells/mm^3^) and those without a delay (350 cells/mm^3^, p = 0.79, Wilcoxon Rank Sum U Test). No CD4 cell count data were missing in the group with a delay in diagnosis. There was no significant difference in median age, proportion of females or proportion of patients with CD4 cell count <200 cells/mm^3 ^between the two groups, although power to detect differences between these groups was low. Table 1 represents descriptive data on the two groups.

**Table 1:** Characteristics of 112 patients diagnosed with HIV infection in a public clinic in rural Haiti*

	**Diagnosis Delayed N (%; 95%CI or IQR)**	**Diagnosis made at first visit N (%; 95%CI or IQR)**	**p value**
Patients (total N = 112)	17	95	-
Median age	28	32	0.44
Female	14 (82.3%; 56.6 – 96.2%)	60 (67.4%; 56.7 – 77.0%)	0.26
Median CD4 count (cells/mm3)	447(250 – 998)	350 (304 – 426)	0.79
CD4 < 200/mm3	2 (11.8%; 1.5 – 36.4%)	19 (22.9%; 14.3 – 33.4%)	0.35
Median no. of days delayed (range)	62 (19–122)	N/A	N/A

*There were no significant differences in these characteristics, although the study was not powered specifically to detect these differences (see methods)

## Discussion

Since 2002, funding has become increasingly available to support HIV treatment in the developing world [13,14]. HIV testing is the critical entry point not only for engagement into treatment and care but also for primary and secondary prevention efforts [15]. Despite the increasing number of HIV treatment programs in resource-poor settings however, uptake of testing is often low and many patients are being diagnosed only when they are profoundly immunosuppressed [16-18]. While few studies have investigated "missed opportunities" and delays in testing in resource poor settings, one Ugandan hospital documented that only half of the inpatients with HIV-related illnesses were offered HIV tests prior to discharge [7] and a South African study documented low uptake of VCT services [19]. Few studies discuss the impact of provider-initiated testing strategies in resource poor settings. In provider-initiated testing, the care provider recommends and offers an HIV test to those individuals who are considered to be at risk of infection. The counseling session is primarily informational and educational, and post-test counseling is provided based on the individuals test result. By building on existing relationships in the health care setting and on health care providers' experience and training, and by offering testing in the context of comprehensive clinical care, provider-initiated testing offers a unique opportunity to increase access to and acceptance of HIV testing.

We evaluated a rural, developing country HIV scale-up program with a provider-initiated strategy for HIV testing, to determine if there were systematic delays in HIV diagnosis because clinicians missed opportunities for HIV testing at the time of clinical encounters. We found that ninety-seven percent of the patients with HIV were diagnosed by the time of their second visit to the clinic. For those whose diagnosis was delayed, a median of only two months passed between their first visit to the newly managed clinic and their HIV diagnosis.

In our setting, pretest counseling is provided by the doctor or nurse seeing the patient in the context of a full medical consultation and other laboratory or radiologic tests as required. Clients wait at the laboratory for their blood sample to be processed and return to the same provider to receive results. Positive HIV test results are hand-carried by laboratory technicians to providers to ensure no loss to follow up. The provider then initiates post-test counseling, and in the event of a positive HIV test, enrolls the patient into clinical care the same day, introducing them to the HIV program social worker and nurse who continue the formal post-test counseling process.

Boucan Carré is a rural, isolated region of Central Haiti, with a population of 40,000 individuals, mostly subsistence farmers, with approximately 23% of the population between the ages of 15 and 49 years. The Partners In Health/Ministry of Health clinic is the only formal healthcare center in the area and at the time of the study was staffed with two physicians, a midwife, a pharmacist, two laboratory technicians and three nurses. Refurbishment activities by PIH began in March 2003 at this center, with full scale up of HIV testing and treatment services available by May of 2003. Prior to this time, the clinic was staffed by one nurse, had frequent stock-outs, no inpatient facilities, no antiretroviral therapy program and HIV testing could only be performed by referral to a center in either the capital city of Port-au-Prince or in the regional capital of Hinche – both three hours away by car. We did not review patients clinic visits prior to refurbishment and support of the clinic in March 2003 because care was extremely limited at that time, records were inconsistent, and because our objective was not to demonstrate missed opportunities for care in the context of a dysfunctional public health clinic (where most opportunities for providing healthcare in the community are likely missed), but to evaluate if there were missed opportunities in the context of a minimal package of services during HIV scale up.

During the time period of review, there were no fixed, specific criteria in place for when to offer HIV testing, however, providers were encouraged to widely offer HIV testing and were trained to identify clinical signs or symptoms of HIV-related disease and opportunistic infections but not to limit testing to only those with suspicion of immunosuppression. None of the patient charts of those who were HIV-infected specifically suggested that the patient had requested the HIV test (as opposed to being initiated by the provider), but it may not be unusual for clients who are interested in HIV testing to present to primary care clinic and to initially ask to be evaluated for minor complaints. Our staff is also trained to identify this possibility and, as mentioned above, to remain open to offering HIV testing broadly.

The results of our study demonstrate that it is possible to have a very low rate of missed opportunities for HIV testing in a high-volume, rural, resource-poor clinic when HIV counseling and testing is integrated into general medical care. This very low rate of missed opportunities occurs, we believe, as a result of a high level of staff awareness and education regarding the importance of considering a diagnosis of HIV infection, and because the same clinician who sees the patient for medical care provides the counseling for HIV testing. This means that HIV voluntary counseling and testing is incorporated into medical care, with no separate wait or visit required by the patient. HIV test results are available within 15 minutes. Comprehensive HIV treatment is available at the clinic.

This study has a number of limitations. It is a retrospective, observational study of patients known to have HIV infection and it does not provide information regarding the HIV status of patients who were not offered testing by the staff. Since the exact number of missed diagnoses of HIV is not known, it is possible that a number of those not tested actually did have HIV. However 55% of patient visits resulted in an HIV test and it is likely that in fact more than 55% of patients were tested, since repeat patient visits often occur in the outpatient department but repeat HIV testing within a period of 9 months is rare. Over a 3-year period, 15,000 HIV tests have been performed at the Boucan Carré health center and the HIV prevalence among those tested of 3% has remained consistent with the prevalence reported in this study – almost double the reported local prevalence. This suggests that only a very small proportion of the untested patients could be infected. Furthermore, those found to have HIV in the clinic had higher CD4 counts at diagnosis than those of individuals diagnosed with HIV in the US or Africa [6,20]. Even if in fact many cases of HIV were missed in Boucan Carre, it is likely that their CD4 counts would have been even higher, since patients who did not have HIV testing would have been less likely to be symptomatic.

Finally, the power to detect clinically meaningful differences between the two groups was low and the lack of differences in Table 1 should not be over-interpreted.

In summary, in contrast to similar studies in US and Africa, we found a very low rate of missed opportunities for HIV testing in a rural, resource-limited clinic setting in Haiti after staff was trained and primary care services were reinforced. This demonstrates the success of a rural HIV testing program that is integrated into primary medical care and initiated by providers. HIV prevention and treatment programs will not achieve success without addressing the urgent need for individuals to be aware of their HIV status in a timely manner and provider-initiated testing can be a successful strategy to address this concern.

The authors wish to thank Partners In Health/Zanmi Lasante staff and patients. This work was supported in part by the National Institute of Allergy and Infectious Disease (T32AI07433, K24AI062476, K23AI063998) and through Partners In Health, the Global Fund to Fight AIDS, Tuberculosis, and Malaria, the Haitian Ministry of Health and numerous private donors. Thanks to Martin Hirsch, MD and Garrett Fitzmaurice, PhD for helpful comments on the manuscript.

## Competing interests

The author(s) declare that they have no competing interests.

## Authors' contributions

LCI designed the study, collected the data and drafted the manuscript. JSM and KAF contributed to the design of the study and helped to draft the manuscript. All authors read and approve the final manuscript.
